# Cobalt–Graphene Catalyst for Selective Hydrodeoxygenation of Guaiacol to Cyclohexanol

**DOI:** 10.3390/nano12193388

**Published:** 2022-09-28

**Authors:** Qichang Guo, Jingbo Mao, Shenmin Li, Jingmei Yin, Yang Lv, Jinxia Zhou

**Affiliations:** College of Environmental and Chemical Engineering, Dalian University, Dalian 116622, China

**Keywords:** cobalt–graphene catalyst, Co single atoms, Co oxide nanoparticles, hydrodeoxygenation, guaiacol, cyclohexanol

## Abstract

Herein, cobalt-reduced graphene oxide (rGO) catalyst was synthesized with a practical impregnation–calcination approach for the selective hydrodeoxygenation (HDO) of guaiacol to cyclohexanol. The synthesized Co/rGO was characterized by transmission electron microscopy (TEM), high-angle annular dark-field scanning TEM (HAADF-STEM), X-ray photoelectron spectroscopy (XPS), Raman spectroscopy, X-ray diffraction (XRD), and H_2_ temperature-programmed reduction (H_2_-TPR) analysis. According to the comprehensive characterization results, the catalyst contains single Co atoms in the graphene matrix and Co oxide nanoparticles (CoO_x_) on the graphene surface. The isolated Co atoms embedded in the rGO matrix form stable metal carbides (CoC_x_), which constitute catalytically active sites for hydrogenation. The rGO material with proper amounts of N heteroatoms and lattice defects becomes a suitable graphene material for fabricating the catalyst. The Co/rGO catalyst without prereduction treatment leads to the complete conversion of guaiacol with 93.2% selectivity to cyclohexanol under mild conditions. The remarkable HDO capability of the Co/rGO catalyst is attributed to the unique metal–acid synergy between the CoC_x_ sites and the acid sites of the CoO_x_ nanoparticles. The CoC_x_ sites provide H while the acid sites of CoO_x_ nanoparticles bind the C-O group of reactants to the surface, allowing easier C-O scission. The reaction pathways were characterized based on the observed reaction–product distributions. The effects of the process parameters on catalyst preparation and the HDO reaction, as well as the reusability of the catalyst, were systematically investigated.

## 1. Introduction

The excessive consumption of fossil resources results in diminishing petroleum supply and severe environmental pollution. As a renewable energy resource, abundant and carbon-neutral biomass has been extensively explored for the production of highly valuable chemicals and biofuels [1]. However, the production of biomass-derived compounds with good selectivity remains challenging because of the complex structures and diverse oxygenic groups in biomass-derived feedstock [2]. Currently, catalytic hydrodeoxygenation (HDO) is considered the most efficient approach for upgrading biomass derivatives, and the development of cost-effective catalysts is key to this process [3]. As a main component of biomass, lignin is a planted polymer composed of phenylpropanoid building units that potentially provide renewable six-ring compounds [4]. Given the complex structure of lignin, guaiacol (GUA; 2-methoxyphenol), which contains two common oxygenate groups in lignin: methoxy (C_aryl_–OCH_3_) and phenolic (C_aryl_–OH) groups, is extensively used as a lignin model compound in catalytic studies of lignin derivatives [5].

HDO of GUA involves combinations of different reactions such as hydrogenation, hydrogenolysis, and dehydration. Different elementary reactions usually occur at different catalytic sites. Typically, the heterogeneous catalysts designed for HDO contain two functions: one is for hydrogen dissociation, while the other is for the C-O activation [6]. To date, a wide range of metal catalysts has been studied for the HDO of GUA. Selective removal of the methoxy group and hydrogenation saturation of the aromatic ring of GUA can produce cyclohexanol (CYHAOL), an important feedstock in the chemical industry [7]. However, the production of CYHAOL from GUA is challenging because versatile hydrogenation processes generate various products. For instance, excessive HDO of GUA forms cyclohexane (CYHA) over numerous catalysts [2,8,9,10,11,12]. Table S1 lists the catalysts identified for the selective HDO of GUA to CYHAOL reported in recent years. Various noble metal catalysts, such as Ru [13] and bimetallic catalysts composed of Ru–Re [14], Ru–Mo [15], Ru–Co [16], and Au–Rh [17], have been widely investigated for the HDO of GUA to CYHAOL. On the other hand, some studies have focused on the development of low-cost transition metal catalysts. Some Ni-based catalysts [18,19,20,21,22,23,24,25] and Co-based catalysts [26,27,28] catalyzed HDO of GUA with CYHAOL as the main product. In particular, the production of CYHAOL in more than 90% yield was achieved over several Co-containing catalysts, including Ru–Co supported on active carbon [16], Ni–Co supported carbon nanotubes [25], and Co supported on TiO_2_ [27]. In this study, we worked to develop a new Co-based catalyst for HDO of GUA with high selectivity to CYHAOL.

Table S2 lists Co-based catalysts used in HDO of GUA. In addition to forming CYHAOL [26,27,28], some Co-based catalysts lead to CYHA [8,9], phenol (Ph) [29,30] and benzene [31,32] products. As shown in Table S2, the Co-based catalysts were reduced with H_2_ [8,9,26,27,29,30,32], NH_3_ [28], or in situ co-pyrolysis with cellulose [31] before use, indicating the reduction treatment of the catalysts was necessary for catalytic activity. For instance, the Co/TiO_2_ catalyst without reduction by H_2_ generated no propylcyclohexanol production in selective HDO of eugenol [27]. When reduced in H_2_ flow at 600 °C for 2 h, it showed excellent HDO activity, achieving >99.9% propylcyclohexanol yield under 1 MPa H_2_, 200 °C for 2 h [27]. Magnetic CoN_x_@NC catalysts synthesized by co-pyrolysis of cellulose and Co(NO_3_)_2_ under an ammonia atmosphere at 650 °C also exhibited good HDO activity for eugenol conversion to propylcyclohexanol [28]. It was noted that the excellent catalytic activity of CoN_x_@NC-650 for propylcyclohexanol formation was mainly ascribed to the CoN_x_ species rather than metallic Co (Co^0^). In addition to transition metals and metal nitrides, carbides are alternative catalytic hydrogenation materials [33]. Metal carbides, such as Co carbide [34], exhibit specific catalytic hydrogenation features in Fischer−Tropsch to olefins reaction. Conventional carbides are a class of interstitial compounds with carbon atoms filled in the crystal structure of the metals. When the metals lose the crystal structures due to excessive dispersion of the metals to nanoparticles, clusters, and even single atoms, the formation of stable metal carbides occurs on carbon composites in crystal structures. In this case, graphene offers many benefits as a carbon support [35]. Graphene is a good support for embedding single metal atoms in the graphene matrix [36]. In addition, the high surface area of graphene promotes the dispersion of active phases, and graphene with good electrical conductivity may be used to modulate the electronic properties of the metal-active sites, thus governing their HDO performance [37].

In this study, the selective HDO of GUA to CYHAOL was investigated over graphene-supported Co catalysts synthesized using a concise and scalable approach. Initial catalyst screening studies demonstrated that Co/reduced graphene oxide (rGO) catalysts are highly suitable for the HDO of GUA to CYHAOL. The structure and composition of the Co/rGO catalyst were characterized through transmission electron microscopy (TEM), high-angle annular dark-field scanning TEM (HAADF-STEM), X-ray photoelectron spectroscopy (XPS), Raman spectroscopy, X-ray diffraction (XRD), and H_2_ temperature-programmed reduction (H_2_-TPR) analysis. The characterization and catalytic experiments revealed the unique catalytic functions of single Co atoms embedded in the graphene matrix and Co oxide nanoparticles on the graphene surface. The effects of heteroatoms in graphene and the conditions of catalyst preparation and reaction, as well as the reusability of the catalyst, were also investigated in detail.

## 2. Materials and Methods

### 2.1. Synthesis of Co-Based Catalysts

rGO was prepared according to a previously reported method [38]. The reduction of graphene oxide (GO) with hydrazine hydrate generated rGO, and GO was prepared with a modified Hummers’ method [38,39]. N-free reduced graphene oxide (rGO*) was prepared by reducing GO with sodium borohydride at 80 °C [40]. Commercial graphene (Gr) was supplied by Aladdin Chemical Reagent Co., Ltd. (China). Cobalt nitrade, ferric nitrade, and nickel nitrade were used to provide the transition metal components of Co, Ni, and Fe, respectively. Comparison experiments were conducted using γ-Al_2_O_3_ (Shandong Filiale of China Aluminum Co., Ltd., Zibo, China; calcined at 500 °C in air before use) and HY zeolite (SAR = 2.6; Wenzhou Huahua Co., Ltd., Wenzhou, China) as supports. The catalysts were prepared with the impregnation–calcination method. The metal loadings were quantitatively confirmed by incipient-wetness impregnation. The typical operations were as follows: First, a cobalt nitrate solution was prepared with an ethanol–water mixture (volume ratio, 1:4); second, Co was dispersed on the support surface with the incipient–wetness impregnation method; the impregnation mixture was vacuum-dried at 50 °C for 12 h; finally, the dried sample was calcined at 500 °C under N_2_ flow in a tube furnace for 2 h (heating rate, 10 °C/min). The prepared catalysts were designated as Co_x_/support, where x represents the amount of Co metal loaded per gram of support (mmol/g). The catalysts were used in the reactions without prereduction treatment. CoO_x_ powder was synthesized for mechanism study through a precipitation method: a cobalt nitrate solution was precipitated with a 0.2 M NaOH solution, and the precipitate was calcined in air at 400 °C.

### 2.2. Characterization

Characterization methods included TEM, HAADF-STEM, XPS, XRD, Raman spectroscopy, low-temperature N_2_ adsorption–desorption, CHNSO elemental analysis, thermogravimetric analysis (TGA), H_2_-TPR, and NH_3_ temperature-programmed desorption (NH_3_-TPD) analysis. Detailed information regarding these methods is provided in the Supplementary Materials.

### 2.3. Catalytic HDO Tests

The HDO of the GUA reactions was conducted in an autocontrol reactor (50 mL; Beijing Century Senlang Experimental Apparatus Co. Ltd., Beijing, China). In a typical run, the reactor was loaded with guaiacol (300 mg), the catalyst (30 mg; without prereduction treatment) and n-dodecane (10 mL), and then pressurized with H_2_ to 1 MPa at room temperature. The reactor was heated to the target temperature and allowed to operate for a specific time. The liquid products were mixed and diluted by ethanol, and then analyzed using an Agilent GC7820 gas chromatograph equipped with a flame ionization detector and SE-30 capillary column (Dalian Zhonghuida Scientific Instrument Co., Ltd., Dalian, China). n-Tetradecane was used as the internal standard. Recycle test of the catalyst was carried out as follows: after the reaction, the catalyst was recovered from the reaction mixture by centrifugal separation, washed with ethanol, and vacuum-dried without calcination for the subsequent run. The results were quantified as GUA conversion, product selectivity, and yield in molar percentage, based on the number of C6 rings in the substrate and products. The overall carbon balance of the products was in the range of experimental error (±3%). The guaiacol conversion (X_GUA_), the Product i selectivity (S_i_), and Product i yield (Y_i_) were calculated as following Equations (1)–(3):

(1) $$\;\;X_{\mathrm{GUA}}\left(\mathrm{mol}\%\right)=\frac{{\left(\mathrm{Moles}\;\mathrm{of}\;\mathrm{GUA}\right)}_{\mathrm{in}}-{\left(\mathrm{Moles}\;\mathrm{of}\;\mathrm{GUA}\right)}_{\mathrm{out}}}{{\left(\mathrm{Moles}\;\mathrm{of}\;\mathrm{GUA}\right)}_{\mathrm{in}}}\times 100\%$$

(2) $$S_{i}\left(\mathrm{mol}\%\right)=\frac{{\mathrm{Moles}\;\mathrm{of}\;\mathrm{Product}}_{i}}{{\left(\mathrm{Moles}\;\mathrm{of}\;\mathrm{GUA}\right)}_{\mathrm{in}}-{\left(\mathrm{Moles}\;\mathrm{of}\;\mathrm{GUA}\right)}_{\mathrm{out}}}\times 100\%$$

(3) $$Y_{i}\left(\mathrm{mol}\%\right)=\frac{{\mathrm{Moles}\;\mathrm{of}\;\mathrm{Product}}_{i}}{{\left(\mathrm{Moles}\;\mathrm{of}\;\mathrm{GUA}\right)}_{\mathrm{in}}}\times 100\%$$

## 3. Results and Discussion

### 3.1. Characterization

#### 3.1.1. Electron Microscope Images

**Figure 1:**
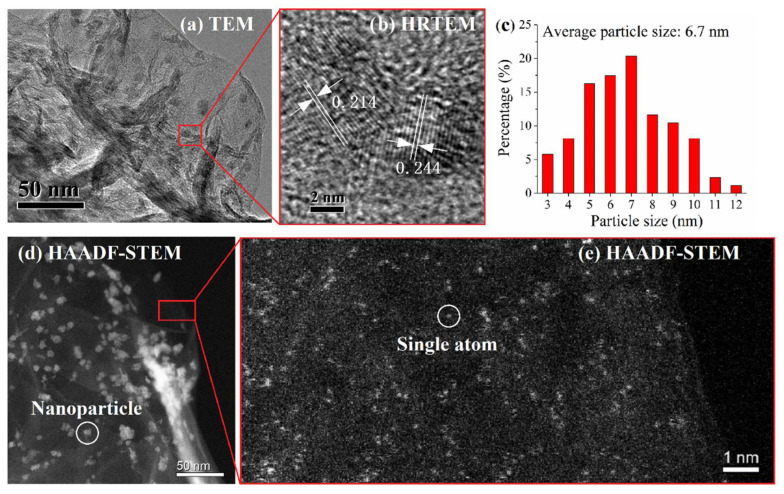
Characteristic results of Co_2.5_/rGO including (**a**) TEM image, (**b**) HRTEM image, (**c**) particle size distribution, and (**d**,**e**) HAADF-STEM images.

The morphology of the Co_2.5_/rGO catalyst was characterized by TEM, HRTEM, and HAADF-STEM (Figure 1). The catalyst shows a homogenous dispersion of nanoparticles (Figure 1a) with an average size of 6.7 nm in the TEM image (Figure 1c). The HRTEM image (Figure 1b) exhibits the typical crystalline morphology of the nanoparticles. The lattice fringes close to 0.213 and 0.244 nm are ascribed to the CoO (200; JCPDS card: 65-2902) and Co_3_O_4_ (311) (JCPDS card: 43-1003) planes, respectively. The lattice fringes of metallic Co were not observed in the HRTEM images. The results indicate the co-existence of various Co oxides in the nanoparticles, which are termed as CoO_x_. Single Co atoms embedded in the graphene sheet were confirmed by HAADF-STEM images (Figure 1d,e). The area marked with a circle is an example of a single Co atom. Figure 1e shows the homogeneous distribution and high density of isolated Co atoms in the graphene nanosheet. The prepared catalyst was calcined at 500 °C in N_2_, and thus, the reserved Co single metal atoms were not physically adsorbed on the graphene sheet; by contrast, they built strong chemical bonding configurations with the rGO sheet. rGO is derived from GO through reduction treatments. After the removal of the oxygenic groups, the *sp*^3^-hybridized C atoms were terminated with H bonds, forming lattice defects on the graphene plane. Chemical bonding of the isolated metal atoms with pristine intact graphene is not easy due to the high chemical stability of the graphene’s honeycomb structure [41]. As confirmed theoretically and experimentally [41,42], the introduction of defective sites in the graphene matrix offers multifarious bonding configurations to guarantee the structural stability of metal atoms. The high density and homogeneous distribution of single Co metal atoms in the rGO sheet (Figure 1e) correspond to the high density and homogeneous distribution of lattice defects in the rGO structure. We named the Co single metal atoms in the graphene matrix as CoC_x_ sites, which are similar to metal carbides.

#### 3.1.2. XPS

**Figure 2:**
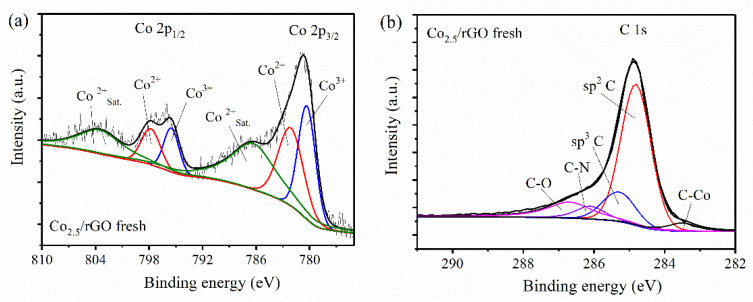
XPS characterization of Co_2.5_/rGO catalyst: (**a**) energy spectrum of Co 2p; (**b**) energy spectrum of C1s.

The XPS results in Figure 2 provide information on the surface elemental constituents and valence state of the Co_2.5_/rGO catalyst. Full-scan XPS survey spectrum (Figure S1 in the Supplementary Materials) shows the predominant presence of C, O, N, and Co elements. The Co^2+^ is characterized by the Co 2p_3/2_ peak at 782.1 eV, Co 2p_1/2_ peak at 798.0 eV, and corresponding shake-up resonances at approximately 787.9 and 803.6 eV [43]. The prominent peaks around 780.3 and 795.3 eV are assigned to the Co 2p_3/2_ and Co 2p_1/2_ peaks of the Co^3+^ configuration with an energy difference of 15 eV [43]. The Co 2p peaks of the bulk cobalt carbide (Co_2_C) appear at 778.4 and 793.4 eV, which are at the binding energy in metallic Co [44]. In the Co_2.5_/rGO catalyst, the Co single-atoms doped in the graphene matrix of rGO formed a cobalt–carbide analog, but no peaks related to the bulk cobalt carbide were observed (Figure 2). A similar phenomenon was reported in a previous study, which showed that the Co XPS of the graphene-supported single Co atoms have two main Co 2p peaks at 780.9 and 796.2 eV with the peak spacing of 15.4 eV and shake-up satellite peaks, indicating that the single Co atoms coordinate with oxidation states [45]. The isolated Co atoms doped in the graphene matrix mainly form multifarious bonding configurations with the surrounding C, and the N and O heteroatoms mediate the Co atoms in oxidation states. Thus, the binding energy of the single Co atoms is larger than that of bulk cobalt carbide. Therefore, the Co^2+^ and Co^3+^ peaks of Co_2.5_/rGO in Figure 2a correspond to isolated Co atoms (CoC_x_) and CoO_x_ nanoparticles.

The C 1s spectrum of the Co_2.5_/rGO catalyst (Figure 2b) splits into several components. The main peak at 284.5 eV corresponds to the sp^2^ C in graphene [46], indicating that most of the C atoms form conjugated honeycomb lattices of graphene. The peak at approximately 285.3 eV is assigned to sp^3^ C, which originates from the lattice defects and edges of the graphene sheets [46]. The peaks from 286 eV to 288 eV correspond to the C−N and C−O bonds [41,46]. The small peak approximately around 283.3 eV is attributed to the carbidic C 1s signal [44], which in turn is attributed to the C atoms bonded with isolated Co atoms of the Co_2.5_/rGO catalyst.

#### 3.1.3. Raman

**Figure 3:**
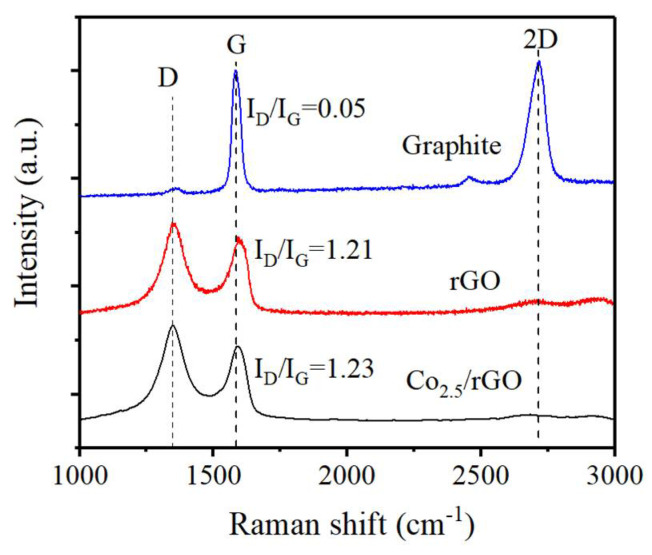
Raman diagrams of graphite, rGO, and Co_2.5_/rGO catalyst.

Figure 3 compares the Raman spectra of graphite, rGO, and Co_2.5_/rGO. Highly ordered graphite has a G-band peak at approximately 1580 cm^−1^, corresponding to the in-phase vibration of the sp^2^ carbon lattice, and a weak D-band peak at approximately 1350 cm^−1^, corresponding to the sp^3^ carbons caused by the defects on the graphite edges [47]. The rGO material contains a significant fraction of the sp^3^ amorphous carbons mainly generated by edges and lattice defects, and thus the rGO sample shows a noticeable D band. The ratio of the peak intensity of the D band to that of the G band (I_D_/I_G_) is inversely proportional to the perfection of the graphene’s honeycomb lattice [47,48]. As shown in Figure 3, the I_D_/I_G_ value of the Co_2.5_/rGO catalyst is close to that of the rGO support, confirming that the isolated Co atoms do not attack the pristine sp^2^ carbon lattice of graphene but are embedded in the defective positions as substitutional or interstitial dopants through the construction of “metal vacancy” heterostructures. Hence, rGO with appropriate lattice defects is an ideal substrate for anchoring isolated Co atoms.

#### 3.1.4. XRD

**Figure 4:**
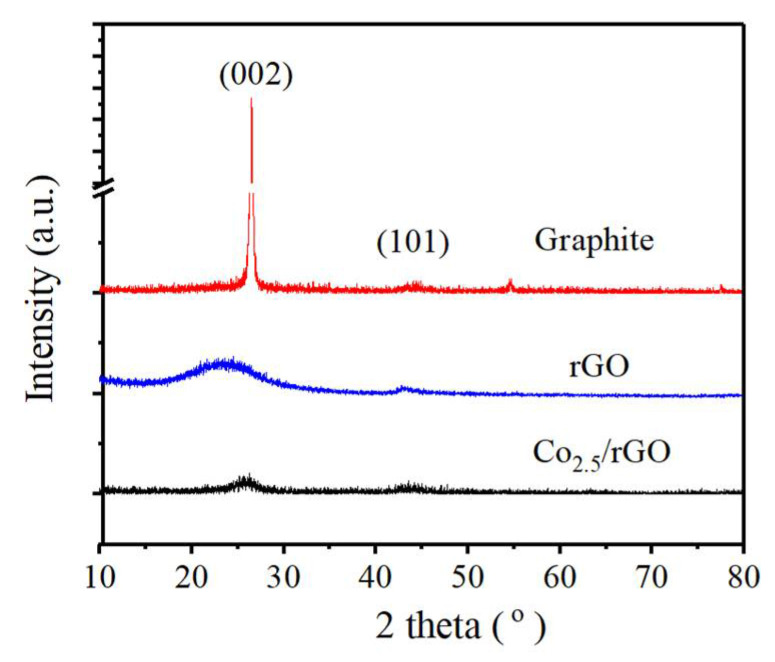
XRD patterns of graphite, rGO, and Co_2.5_/rGO.

The crystal structures of graphite, rGO, and Co_2.5_/rGO were investigated through XRD analysis (Figure 4). Graphite has a sharp peak at a 2 theta of 26.4°, corresponding to the (002) plane of graphite (JCPDS PDF card: 41-1487). The XRD pattern of rGO shows trace amounts of graphite (002) diffraction signals with a widened peak and a low-degree-shift 2 theta, showing that rGO has a multilayer structure with increased lattice dimensions. The XRD peaks of the Co_2.5_/rGO sample show a slightly enhanced and high-degree-shift 2 theta of the (002) plane compared with that of rGO, which is caused by the stacking thickness of the graphene layers during the preparation of the catalyst. In addition to the graphitic diffraction, the XRD pattern of the Co_2.5_/rGO catalyst shows no diffraction signals corresponding to Co-related diffraction, indicating the small crystalline sizes and weak crystallinity of the Co species. This finding is in accordance with the TEM images (Figure 1a).

#### 3.1.5. H_2_-TPR

**Figure 5:**
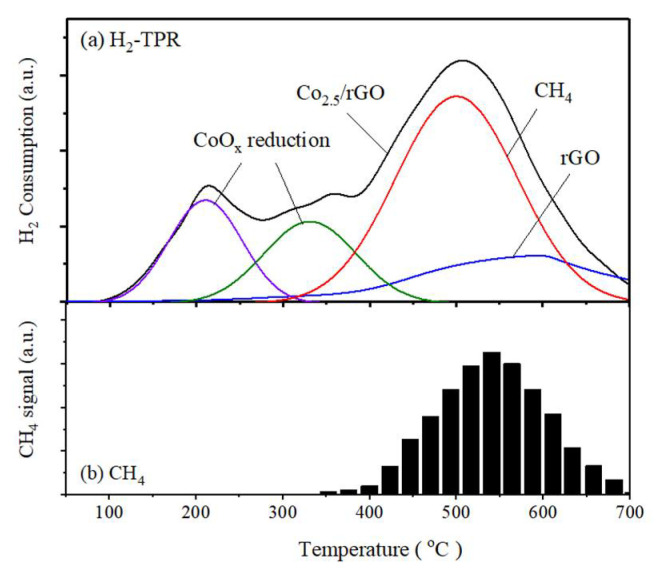
H_2_-TPR patterns of Co_2.5_/rGO (**a**) and CH_4_ in the TPR exhaust gas (**b**).

The TPR patterns of the Co_2.5_/rGO catalyst are shown in Figure 5. rGO has certain oxygenic groups on its surface and periphery [49]. The hydrogenolysis of the –OH, –O–, and –COOH groups involves H_2_ consumption starting at 400 °C. In the Co_2.5_/rGO catalyst, rGO and Co oxides consume H_2_. Moreover, according to the GC analysis, CH_4_ exists in the TPR exhaust gas (Figure 5b). Thus, the H_2_ consumption of the Co_2.5_/rGO sample includes the decomposition of oxygenic groups from rGO, reduction of Co oxides, and formation of CH_4_. The multipeak reduction of Co oxides corresponds to the step–step reduction of Co_2_O_3_/Co_3_O_4_ to CoO and CoO to metallic Co [50]. A mixture of metallic Co powder (pre-reduced from CoO_x_ powder) and rGO cannot generate CH_4_ at a temperature of approximately 530 °C; therefore, the formation of CH_4_ is a representative feature of the Co_2.5_/rGO sample during the H_2_-TPR test. We speculate that the single Co atoms embedded in the graphene plane catalyze the bonded C atoms of rGO to form CH_4._

#### 3.1.6. Elemental Analyses

The three graphene materials, rGO, rGO*, and Gr, have similar specific surface areas but different elemental compositions (Table 1). The O atoms mainly come from the oxygenic groups, such as –OH and –COOH. The H atoms originate from the dangling H atoms for the termination of the dangling bonds of the graphene sheet edges, defects, and some groups. The rGO and rGO* materials have similar C, H, and O contents, but rGO contains 3.7 mol% N atoms. N was incorporated into the rGO network through the hydrothermal reduction of GO using hydrazine hydrate and ammonia as reducing reagents [51]. The comparison of rGO with Gr shows that rGO contains more H than Gr dose. More H indicates more lattice defects because the C dangling bonds are terminated by the H atoms.

### 3.2. Catalyst Investigation

#### 3.2.1. Possible Reaction Pathway

**Scheme 1:**
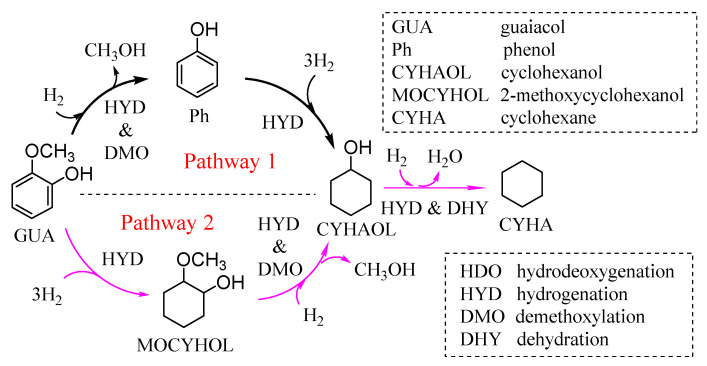
Possible reaction pathways in the HDO of GUA over the Co/rGO catalyst.

GUA contains C_aryl_–OH, C_aryl_–OCH_3,_ and C_aryl_ O–CH_3_ bonds and an aromatic ring in its structure. The catalytic conversion of GUA may generate a wide range of products over metal catalysts, including those formed without oxygen removal, partial oxygen removal, and complete oxygen removal. These products are generated by different reaction pathways, such as hydrogenation (HYD), demethoxylation (DMO), and dehydration (DHY). The possible reaction pathways for the HDO of GUA were explored based on the product distributions. As shown in Scheme 1, the main pathway involves the cleavage of C_aryl_–OCH_3_ bond to phenol (Ph) through HYD and DMO followed by the saturation of the aromatic ring to CYHAOL through HYD; the other involves the saturation of the aromatic ring to 2-methoxycyclohexanol (MOCYHOL) through HYD and then the cleavage of C_alkyl_–OCH_3_ bond to CYHAOL. The DHY of CYHAOL generates a CYHA by-product.

#### 3.2.2. Catalyst Screening Study

The catalysts’ performance for HDO of GUA depends on two types of active sites: one is the metal-like site that readily activates hydrogen, while the other type of site nearby, such as an acid site, is responsible for the C-O bond activation [6,52]. For screening the functional catalyst, Fe, Co and Ni supported on rGO, and Co supported on different supports were tested in HDO of GUA. The catalytic performance of the prepared catalysts is presented in Table 2. Fe, Co, and Ni are eight-group elements with outer electronic structures of 3d^6^4s^2^, 3d^7^4s^2^, and 3d^8^4s^2^, respectively, and the three corresponding catalysts have similar specific surface areas but different catalytic activities (Table 1, entries 1–3). When the reaction occurs over the Co_2.5_/rGO catalyst at 200 ℃ and 1.0 MPa H_2_ pressure for 2 h, GUA is completely converted, and the yield of CYHAOL reaches 93.2 mol%. The Ni_2.5_/rGO catalyst achieves 90.1 mol% conversion with 62.4 mol% and 35.1 mol% selectivity to CYHAOL and MOCYHOL, respectively. Ni catalysts usually catalyze total HYD to MOCYHOL as a major side reaction, especially at lower reaction temperatures [26]. The Fe_2.5_/GO catalyst only converts 1.8 mol% of GUA. CYHA as a by-product is rarely detected in the reaction catalyzed by Co_2.5_/rGO; therefore, Co is the most suitable transition metal to be supported on rGO for HDO reaction.

The support material is another decisive factor for determining catalyst activity. Compared with the Co_2.5_/rGO catalyst, the Co_2.5_/Al_2_O_3_ and Co_2.5_/HY samples (Table 2, entries 4 and 5) show much lower activities. In general, a pristine metal species is an active component in the hydrogenation reaction; thus, the catalysts are prereduced or originally contain metals before they are used in GUA hydrogenation [13,14,15,16,17,18,19,20,21,22,23,24,25,26]. In this study, all the catalysts were used without prereduction treatments. Co in the Co_2.5_/Al_2_O_3_ and Co_2.5_/HY catalysts is in an oxide state, and thus, the catalysts exhibit extremely low activities because of insufficient metallic Co. However, the Co species in Co_2.5_/rGO contains no metallic Co according to the HRTEM, XPS, and XRD characterization results, and the catalyst was used without prereduction treatment. Co_2.5_/rGO was directly exposed to air during collection and weighing. Therefore, the active sites of Co_2.5_/rGO are different from the frequently used type of catalysts requiring reduction treatment. Transition metal carbides, which are prepared by incorporating carbon atoms into the lattices of transition metals, have been demonstrated as promising catalysts for biomass conversions, especially in the C-C, C-O-C, and C-O-H bonds cleavage reactions [53]. We speculate that the catalytic component of Co_2.5_/rGO for hydrogenation originates from a Co–carbide analog (CoC_x_) formed by embedding Co single atoms in the graphene matrix. The poor reaction result over the physical CoO_x_ + rGO mixture (Table 2, entry 6) confirms the critical role of the CoC_x_ sites in hydrogenation catalysis. Thus, rGO as a support is involved in the construction of the CoC_x_ sites, whereas Al_2_O_3_ and HY support lack such functions.

The above results indicate that using rGO as a support is one of the decisive factors in determining the performance of the Co_2.5_/rGO catalyst. According to the elemental analysis results (Table 1), rGO^*^ is an N-free material, and rGO contains 3.7 mol% N. Co_2.5_/rGO^*^ (Table 2, entry 7) achieves 85.0 mol% conversion and 59.5 mol% CYHAOL yield, manifesting that the doped N atoms are not an indispensable factor in catalytic function. However, the improved activity of the Co_2.5_/rGO catalyst as compared with that of Co_2.5_/rGO^*^ confirms a positive role of N doping in graphene. Transition metal nitrides have been explored in HDO conversions [28]. Moreover, the N atoms merged within the graphene matrix disrupt the electronic neutrality of adjacent carbon atoms [54,55], which manipulate the electronic status of CoC_x_ and CoO_x_ in higher electronic density. This effect improves the activity of the Co_2.5_/rGO catalyst. The O functional groups of rGO may improve the dispersion and stability of the supported CoO_x_ nanoparticles through an anchoring effect during impregnation and calcination. In brief, rGO with appropriate amounts of N and O heteroatoms is a suitable graphene material as a support.

In order to confirm the hypothesis that the hydrogenation function of Co_2.5_/rGO is provided by CoC_x_, the Co_2.5_/Gr catalyst prepared with a commercial graphene (Gr) was investigated for clarity. The conversion and CYHAOL yield over Co_2.5_/Gr (Table 1, entry 8) is much less than that over Co_2.5_/rGO. The catalyst-specific surface area of Co_2.5_/Gr is 341 m^2^·g^-1^, and the average particle size of CoO_x_ nanoparticles on Gr surface (Figure S2a) is 6.5 nm, which indicates that the low activity of Co_2.5_/Gr is not caused by the small specific surface area and the low dispersion of CoO_x_ components. Supposing the hydrogenation function of Co_2.5_/rGO derives from the in situ reduction of CoO_x_ nanoparticles to metallic Co during reaction, then Co_2.5_/Gr should be as good—or as bad—as Co_2.5_/rGO; therefore, some critical factors determine the catalysts’ performance. The biggest difference between rGO and Gr is that rGO can anchor more single-metal atoms than Gr does. The evidence is shown in the HAADF-STEM image of Co_2.5_/rGO. (Figure 1e) shows the high-density distribution of the isolated Co atoms in the graphene matrix, while the number of the isolated Co atoms decreases considerably in the image of Co_2.5_/Gr (Figure S2b). The more Co single atoms in the graphene matrix, the more Co–carbide analog (CoC_x_) the catalyst contains. Transition metal carbides have been compared to platinum group metals, showing similar catalytic properties, which means that they could be promising catalysts for HDO reactions [52]; therefore, Co_2.5_/Gr exhibits weak catalytic activity due to the low population of the CoC_x_ sites.

**Scheme 2:**
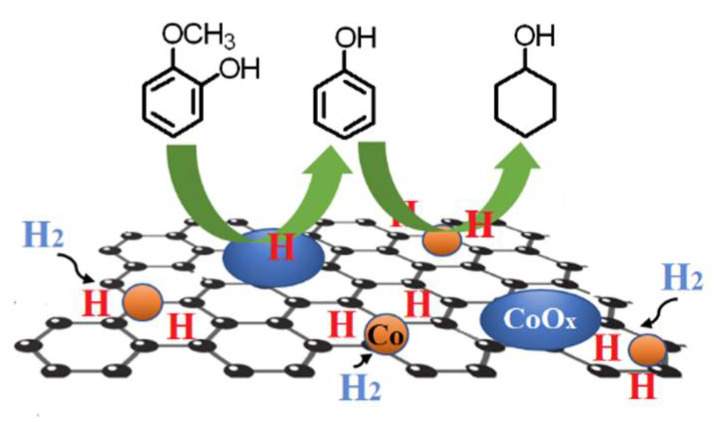
Possible reaction mechanism of HDO of GUA over the Co_2.5_/rGO catalyst with Ph as the intermediate.

The data in entry 6 of Table 2 show that CoO_x_ fails to conduct the HDO of GUA without the participation of CoC_x_; however, the CoO_x_ nanoparticles on rGO surface may improve the ability of the Co_2.5_/rGO catalyst to break C_aryal_–OCH_3_ bond. HDO was proposed to need a bifunctional catalyst where acid sites are required for C–O bond activation, allowing easier C–O scission [6]. CoO_x_ contains acidity according to the NH_3_-TPD result (Figure S3a) and can adsorb the oxygenic groups in a GUA molecule through acid–base-pairing interactions, facilitating the cleavage of the C–O bond. Therefore, CoO_x,_ in combination with CoC_x,_ plays a role in establishing the metal−acid bifunction of the catalyst. A similar bifunctional catalyst was established for HDO reaction based on molybdenum carbide and oxide [56]. When Ph and CYHAOL are used as feedstock and the reaction is run at 200 °C and 1 MPa H_2_ for 2 h, Ph is completely converted to CYHAOL over the Co_2.5_/rGO catalyst, whereas CYHAOL is almost not converted. Cleavage of the C_aryl_–OH bond needs strong acidic components such as HZSM-5 zeolite [2,28], while the mild acid of CoO_x_ appropriately avoids cleaving the C_aryl_–OH bond. In this reaction, especially carried out in milder conditions, the basicity of support can affect the selectivity of bond breaking [6,57,58]. The presence of base promotes the demethoxylation step and suppresses the unselective C-O dissociation [57]. The basicity of CoO_x_ is not obvious according to the CO_2_-TPD result (Figure S3b), but N doping incorporates basic sites in the graphene texture [26], which potentially favors the C_aryl_–OCH_3_ bond cleavage, making the Co_2.5_/rGO catalyst has good selectivity to CYHAOL. Scheme 2 shows the main catalysis in the selective HDO of GUA to CYHAOL over the Co_2.5_/rGO catalyst, in which Ph is used as the intermediate. H_2_ dissociates to active H species on the CoC_x_ sites; the C_aryl_–OCH_3_ bond of GUA is activated by the oxophilic acid sites of CoO_x_ and dissociated by active H. The metal–acid bifunction of the Co_2.5_/rGO catalyst in the HDO of GUA to CYHAOL is attributed to the unique synergy between the CoC_x_ sites and the acid sites of the CoO_x_ nanoparticles. The Co_2.5_/rGO catalyst selectively saturates the aromatic ring and dominantly cleaves C_aryl_–OCH_3_ bond in the GUA. These processes result in a good performance in CYHAOL production.

#### 3.2.3. Optimization of Catalyst Preparation Conditions

**Figure 6:**
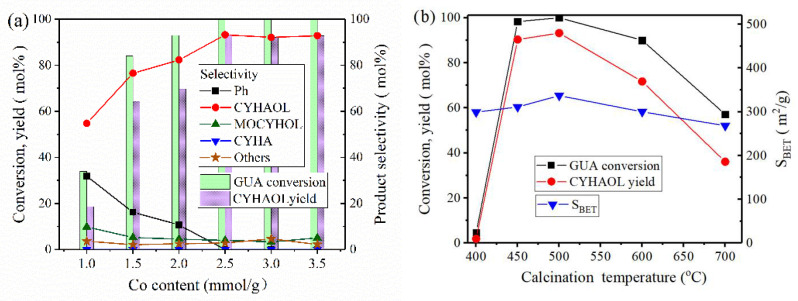
Influence of catalyst preparation conditions on HDO of GUA reactions: (**a**) Co loading amounts; (**b**) catalyst calcination temperature (reaction conditions: 300 mg of GUA in 10 mL of n-dodecane; catalyst loading = 30 mg; 200 °C; 1 MPa H_2_; 2 h).

The effects of the Co loading and calcination temperature on the Co/rGO catalysts were studied. The results are shown in Figure 6. Co/rGO catalysts with different Co loadings were prepared for the HDO of GUA (Figure 6a). When the Co loading increases from 1.0 mmol/g to 2.5 mmol/g, the yield of CYHAOL constantly increases and reaches 93.2 mol% at 2.5 mmol/g; further increase in Co loading has little contribution to the CYHAOL yield. Figure 6b shows the results obtained for the Co_2.5_/rGO catalysts at different calcination temperatures in N_2_. The conversion of GUA and the yield of CYHAOL shows volcano-like shapes when the calcination temperature is increased from 400 °C to 700 °C, achieving maximum values at 500 °C. The Co_2.5_/rGO catalyst calcinated at 400 °C in N_2_ only leads to 4.6 mol% conversion of GUA. When the calcination temperature is above 500 °C, the aggregation of nanoparticles becomes obvious, as evidenced by the low specific surface areas of the samples calcined at 600 °C and 700 °C. The highest efficiency for the HDO of GUA was obtained using a catalyst with a loading of 2.5 mmol Co per gram of rGO at a calcination temperature of 500 °C.

Blanco et al. [26] prepared the Co/GOr and Co/GOr-N catalysts by wet impregnation of Co over reduced graphene oxide undoped and doped with N, calcination in N_2_ at 350 °C and reduction under H_2_ at 300 °C. As evidenced by the XRD pattern and CO chemisorption of the catalysts, the hydrogenation sites of Co/rGOr and Co/rGOr-N are metallic Co (Co^0^) components. In our study, the Co_2.5_/rGO catalyst calcinated at 500 °C in N_2_ and used without H_2_-reduction pretreatment exhibits good activity. Metallic oxides or nitrates on carbonaceous supports may form metals by carbothermal reduction during calcination in inert gases. For example, NiFe alloy nanoparticles were prepared by calcination of a cellulose filter paper impregnated with Fe and Ni nitrates at 800 °C for 2 h under N_2_ [59]. The Co_2.5_/rGO catalyst calcinated at 500 °C in N_2_ does not contain metallic Co according to the XRD pattern and the lattice fringes in the HRTEM image of the catalyst. Therefore, the CoC_x_ sites of Co_2.5_/rGO serve as the activity sites. The low activity of the Co_2.5_/rGO catalyst calcined at 400 °C in N_2_ manifests that the calcination temperature at 400 °C or below cannot provide sufficient energy for the formation of the CoC_x_ sites, so the Co/GOr and Co/GOr-N catalysts calcinated at only 350 °C [26] can hardly contain the CoC_x_ sites.

#### 3.2.4. Effects of Reaction Conditions

**Figure 7:**
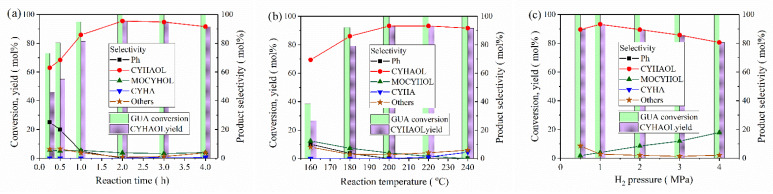
Influence of (**a**) reaction time, (**b**) reaction temperature, and (**c**) H_2_ pressure on the HDO of GUA (Reaction conditions: 300 mg of GUA in 10 mL of n-dodecane, catalyst loading = 30 mg. Other conditions: (**a**) 200 °C, 1 MPa H_2_; (**b**) 1 MPa H_2_, 2 h; (**c**) 200 °C, 2 h).

Figure 7 shows the variations in GUA conversion and product distribution among different reaction conditions. As reaction time proceeds, GUA conversion rate increases rapidly and reaches 100 mol% at 2 h. Selectivity to the Ph intermediate product gradually decreases over time after the initial accumulation, accompanied by an increase in selectivity to CYHAOL. This result shows that Ph is an intermediate progressing toward CYHAOL. An increase in reaction temperature from 160 °C to 200 °C increases the conversion rate of GUA and yield of CYHAOL. When the reaction approaches the 100 mol% conversion, the improved reaction temperature slightly decreases the CYHAOL yield, indicating that CYHAOL is relatively stable over the temperature range from 200 °C to 240 °C. With increasing H_2_ pressure from 0.25 MPa to 4.0 MPa, the CYHAOL yield reaches 93.2 mol% at 1.0 MPa and then decreases gradually with pressure, and selectivity to MOCYHOL increases. These effects indicate that high H_2_ pressure intensifies the HYD saturation of the aromatic ring to form MOCYHOL. Theoretically, MOCYHOL should proceed toward CYHAOL through the cleavage of C_alkyl_-OCH_3_ bond. However, the fully hydrogenated MOCYHOL is more stable than Ph, and the higher steric hindrance of MOCYHOL restrains the cleavage of C_alkyl_-OCH_3_ bond. Therefore, the GUA–Ph–CYHAOL pathway is much more conducive to producing MOCYHOL than the GUA–MOCYHOL–CYHAOL pathway, and a lower H_2_ pressure facilitates CYHAOL formation.

#### 3.2.5. Catalyst Recycle Study

**Figure 8:**
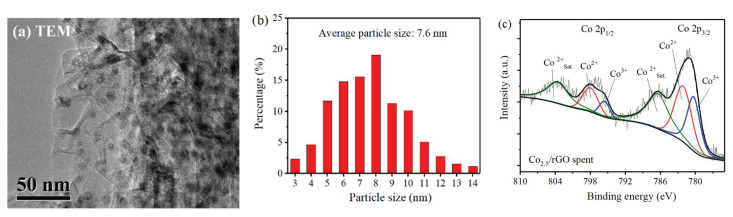

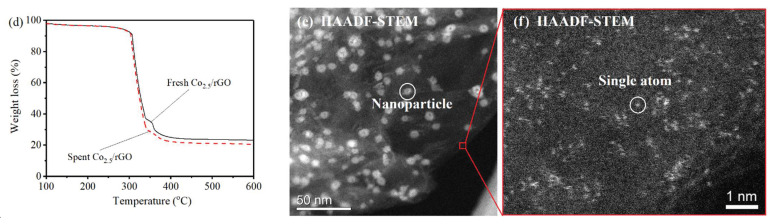
Characterization results of the spent Co_2.5_/rGO catalyst: (**a**) TEM image; (**b**) particle size distribution; (**c**) energy spectrum of Co 2p in XPS result; (**d**) TG/DTA image; (**e**,**f**) HAADF-STEM images.

Catalyst stability is a critical factor in developing conversion processes industrially. Sintering and leaching of metal components are major problems for liquid-phase HDO reactions [60]. Further, coking on the catalyst and structural degradation leads to the deactivation of the catalysts [60]. Figure 8 summarizes the characterization results of the spent Co_2.5_/rGO catalyst. The TEM image of the spent Co_2.5_/rGO catalyst shows the homogenous dispersion of nanoparticles (Figure 8a) with a slightly larger average size of 7.6 nm (Figure 8b) as compared to that of a fresh one (6.7 nm). Fresh Co_2.5_/rGO catalyst contains no metallic Co (Figure 2a) and is used without prereduction treatment. A hypothesis states that Co_2.5_/rGO catalysts undergo in situ reduction to form metallic Co. However, the XPS spectra of the spent catalyst (Figure 8c) show no metallic Co, thereby confirming that the active sites for activating H_2_ is CoC_x_ rather than metallic Co. Figure 8d shows the TG curves of the fresh and spent Co_2.5_/rGO catalysts. The weight loss rates mainly relate to the burning of rGO at approximately 310 °C, and the coke, if present, cannot be identified because it burnt with rGO together. However, a careful comparison of the two curves found that the weight loss of the spent catalyst is about 3% higher than that of the fresh one, which is caused by coke deposition on the spent catalyst. Figure 8e,f show the high density of isolated Co atoms in the graphene nanosheet of the spent catalyst.

**Figure 9:**
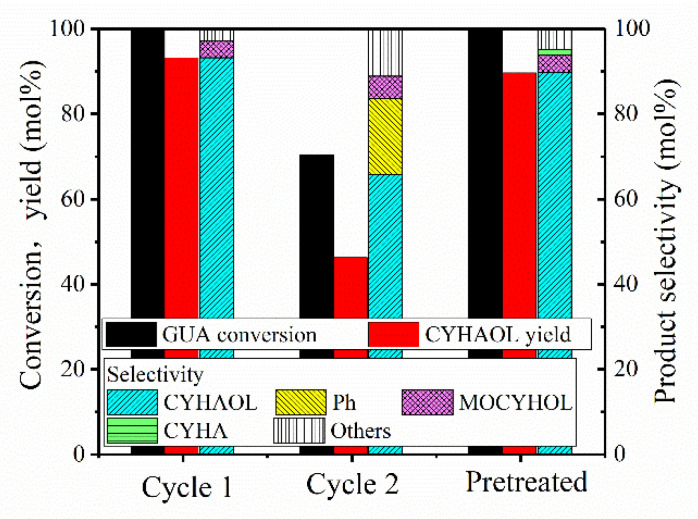
Reuse of Co_2.5_/rGO in HDO of GUA to CYHAOL (reaction conditions: 300 mg of GUA in 10 mL of n-dodecane; catalyst loading = 30 mg; 200 °C; 1 MPa H_2_; 2 h).

The reusability of the Co_2.5_/rGO catalyst was investigated during the HDO of the GUA (Figure 9). The catalyst recycling experiment was performed without regeneration. The conversion rate of GUA drops to 70.4 mol% in the repeated test (cycle 2), indicating the deactivation of the Co_2.5_/rGO catalyst. One concern regarding the causes of deactivation is that single Co atoms in the graphene matrix may move and aggregate during the thermodynamic process of a reaction, which leads to the loss of CoC_x_ sites. A model reaction was performed to exclude this possibility. The fresh Co_2.5_/rGO catalyst was first processed under the following conditions: 10 mL of n-dodecane, 30 mg of the catalyst, 200 °C, and 1 MPa H_2_ for 2 h. Then, 300 mg of GUA was added to the reactor, and the reaction proceeded under the same conditions. The result of “Pretreated” in Figure 9 shows that the catalyst maintains its activity, indicating that the single Co atoms embedded in the rGO lattice are stable under the reaction conditions, in accordance with the image of HAADF-STEM (Figure 8f). Thereby, coking is the main reason for the deactivation of the catalyst. Coking was also presented in previous studies on the catalytic HDO of GUA [21,60,61,62,63].

## 4. Conclusions

The catalytic HDO of GUA, a phenolic model compound of biomass lignin pyrolysis products, was investigated over Co/rGO catalysts synthesized through a practical impregnation–calcination approach. A series of characterization results revealed that the prepared Co/rGO catalyst contains single Co atoms embedded in the graphene matrix and CoO_x_ nanoparticles on the graphene surface. The isolated Co atoms formed stable metal carbide analogs (CoC_x_) with rGO when the catalyst was calcined at 500 °C in N_2_. The graphene texture is a decisive factor in determining the catalyst performance. rGO with proper N heteroatoms and lattice defects is a suitable material for catalyst fabrication. The optimized Co/rGO catalyst without prereduction treatment led to the complete conversion of GUA with 93.2% yield to CYHAOL under mild reaction conditions (200 °C and 1.0 MPa H_2_ pressure for 2 h). The catalytic HDO of GUA to GYHAOL mainly occurred through the pathway using phenol as the intermediate. The Co/rGO catalyst possesses metal–acid bifunctional characteristics, by which the CoC_x_ sites readily activate H_2_ and provide active H, and the CoO_x_ nanoparticles provide acid sites that are required to activate the C–O bond, allowing easier C–O scission by active H. The isolated Co atoms of the Co/rGO catalyst were stable under the reaction conditions of 200 °C and 1.0 MPa H_2_, which emphasized the feasibility of constructing the highly dispersed metal–carbide species through embedding single metal atoms in the graphene matrix.

## Figures and Tables

**Table 1:** Property of graphene materials.

Entry	Support	*S*_BET_ (m^2^·g^−1^)	Element Composition (mol%)				Molar Ratio
			C	H	O	N	C:H:O:N
1	rGO	506	70.3	16.0	10.0	3.7	100:23:14:5
2	rGO^*^	516	70.1	17.6	12.2	0	100:25:17:0
3	Gr	583	84.7	7.2	7.1	1.0	100:9:8:1

**Table 2:** HDO of GUA over the prepared catalysts ^a^.

Entry	Catalyst	*S*_BET_ (m^2^·g^−1^)	Conversion (mol%) GUA	Yield (mol%) CYHAOL	Selectivity (mol%)				
					Ph	CYHAOL	MOCYHOL	CYHA	Others ^b^
1	Co_2.5_/rGO	337	100	93.2	-	93.2	4.0	-	2.8
2	Ni_2.5_/rGO	338	90.1	56.7	-	62.4	35.1	0.6	1.9
3	Fe_2.5_/rGO	328	1.8	0.6	38.8	51.5	-	-	9.7
4	Co_2.5_/Al_2_O_3_	413	-	-	-	-	-	-	-
5	Co_2.5_/HY	572	2.2	1.4	32.2	63.2	-	-	4.6
6	CoO_x_+rGO ^c^		2.7	-	-	-	-	-	-
7	Co_2.5_/rGO*	349	85.0	59.5	15.4	70.0	4.2	-	10.4
8	Co_2.5_/Gr	341	20.5	7.8	46.7	38.0	7.7	-	7.6

^a^ Reaction conditions: 300 mg of GUA in 10 mL of n-dodecane; catalyst loading = 30 mg (10 wt.% with respect to GUA); 200 °C; 1 MPa H_2_; 2 h; GUA–guaiacol, CYHAOL–cyclohexanol, Ph–phenol, MOCYHOL–2-methoxycyclohexanol, CYHA–cyclohexane; ^b^ unidentified and unlisted product mixtures were labeled as “Others”; ^c^ 30 mg of CoO_x_ and 30 mg of rGO.

## Data Availability

Not applicable.

## Supplementary Materials

The following supporting information can be downloaded at: https://www.mdpi.com/article/10.3390/nano12193388/s1, including the characterization description; Figure S1: The full-scan XPS survey spectrum of the Co_2.5_/rGO catalyst; Figure S2: Characterization results of the Co_2.5_/Gr catalyst: (a) TEM image and particle size distribution, (b) HAADF-STEM image; Figure S3: The NH_3_-TPD result of the CoO_x_ powder; Table S1: Catalysts for hydrodeoxygenation of guaiacol to cyclohexanol; Table S2: Co-based catalysts used in hydrodeoxygenation of guaiacol. References [8,9,13,14,15,16,17,18,19,20,21,22,23,24,25,26,27,28,29,30,31,32] are cited in the Supplementary Materials.
