# Supplementary material for: Cobalt–Graphene Catalyst for Selective Hydrodeoxygenation of Guaiacol to Cyclohexanol
Source: Nanomaterials (Basel). 2022 Sep 28;12(19):3388. doi: 10.3390/nano12193388 (PMC9565367; doi:10.3390/nano12193388)
Supplement: Supplementary file 1 [file nanomaterials-12-03388-s001.zip › nanomaterials-1919200-supplementary.pdf]

nanomaterials

Supplementary Materials

Cobalt–Graphene Catalyst for Selective Hydrodeoxygenation of Guaiacol to Cyclohexanol

Qichang Guo, Jingbo Mao, Shenmin Li, Jingmei Yin, Yang Lv and Jinxia Zhou *

College of Environmental and Chemical Engineering, Dalian University, Dalian 116622, China
* Correspondence: zhoujinxia@dlu.edu.cn; Tel.: +86-411-87403214; Fax: +86-411-87402449

1. Characterization

High-resolution transmission electron microscopy (HRTEM) images were recorded on Tecnai G2 F30 microscope (FEI Co., Hillsboro, OR, USA). High-angle annular dark-field scanning transmission electron microscopy (HAADF-STEM) images were obtained on JEOL JEM-ARM200F STEM/TEM (JEOL, Tokyo, Japan) equipped with a CEOS probe corrector, with a guaranteed resolution of 0.08 nm. The samples were dispersed in ethanol and deposited onto copper grids coated with a thin holey carbon film. X-ray photoelectron spectroscopy (XPS) were characterized on ESCALAB-250 (Thermo Scientific Co., Waltham, MA, USA; Al Kα monochromatic Xray source (E=1486.6 eV), and 50 eV pass energy). The XPSPEAK 4.1 program (XPSPEAK, version 41; Software for the analysis of XPS spectra; Raymund Kwok: Hong Kong, China, 1994.) was used for mathematical analysis of the peaks in the spectra. The binding energies were aligned based on the graphitic carbon (sp^2 carbon) C 1s peak of graphene at 284.5 eV, which corresponds to the main signal of the deconvoluted high-resolution C1s XPS spectra of the catalyst sample. X-ray powder diffraction (XRD) patterns were recorded at room temperature on Rigaku SmartLab 9kW (M/s. Rigaku Co., Tokyo, Japan) X-ray diffractometer using Cu Kα radiation (λ = 1.5406 Å) with a scan range of 2°–80° at 40 kV and 30 mA. Raman spectroscopy was performed on a Renishaw InVia Raman microscope (Renishaw Co., UK) with a solid-state laser (excitation at 532 nm, 0.3 MW and 600 s). The temperature programmed reduction (TPR) analysis was conducted with AutoChem II 2920 Chemisorption Analyzer (Micromeritics Instruments Co., Norcross, GA, USA) fitted with a thermal conductivity detector (TCD). For each test, 20 mg of the sample was dried in nitrogen at 200 °C for 1 h and then reduced in 5 vol.% H_2/Ar gas mixture (30 mL/min) at a linear heating rate of 10 °C/min in the temperature range of 50 °C to 800 °C. The exhaust gas after removal of moisture was monitored by a TCD detector for testing H_2 consumption amount, followed by component analysis with gas chromatography (Agient 7820with a TCD detector and an FID detector; Agilent Technologies Co. Ltd., Santa Clara, CA, USA). The NH_3-temperature programmed reduction investigation (NH_3TPD) was also conducted with AutoChem II 2920 Chemisorption Analyzer. Surface area measurement with nitrogen adsorption was performed with Micromeritics ASAP 2020 (Micromeritics Instruments Co., USA). Specific surface area (S_{BET}) was determined by BET equation (P/P^0 = 0.05–0.3). Thermogravimetric analysis (TGA) results were acquired using a TGA/SDTA851e instrument (Mettler Toledo Co. Ltd., Columbus, OH, USA). Samples were heated in a flow of dried air gas (20 mL min^{-1}) from room temperature to 700 °C with a ramp rate of 10 °C min^{-1}. Mass spectroscopy was performed on GC–MS system (HP Agilent 6890N-5973N; Agilent Technologies Co. Ltd., USA) for compound structure analysis. The contents of C, H, O, and N in graphene materials were obtained with Elemental Analyzer Vario EL cube (Elementar Co., Langenselbold, Germany).

2. Tables

Table S1. Catalysts for hydrodeoxygenation of guaiacol to cyclohexanol [a].

Entry	Catalyst	Reduction condition	Reaction conditions				X_{GUA} mol%	Y_{CYHAOL} mol%	Ref.
			Temp. °C	Time h	Solvent	H donator (H_2 pressure)			
1	Ru/TiO_2	H_2/250°C	240	1	1,4-dioxane	H_2(1.0MPa)	71.7	51	[13]
2	Ru/C	H_2/250°C	200	5	2-propanol	2-propanol	>99	70.2	[14]
3	Ru-MnO/AMWCNTs	H_2/400°C	200	3.33	decalin	H_2(2MPa)	99.4	85.8	[15]
4	Ru-Co/C-600	H_2/600°C	200	1.5	n-decane	H_2(1MPa)	100	~94	[16]
5	AuRh/TiO_2	H_2/300°C	280	6	-	H_2(4MPa)	94	58.3	[17]
6	Ni/ZrO_2-CeO_2	H_2/450°C	220	3	decane	H_2(2MPa)	100	~85	[18]
7	NP-Ni (MA)	Vacuum	180	4	H_2O	H_2(2MPa)	99.7	89.8	[19]
8	Ni/SiO_2-Al_2O_3	H_2/460°C	260	3	decalin	H_2(5MPa)	98.7	55	[20]
9	NiCNTm	H_2/350°C	300	2	-	H_2(5MPa)	100	~60	[21]
10	NiZr–I/CMK-3	H_2/450°C	300	8	hexadecane	H_2(5MPa)	100	47.9	[22]
11	NP-NiMnO_2	N_2 protection	150	12	H_2O	H_2(0.5MPa)	100	75	[23]
12	NiCo/γ-Al_2O_3	H_2/400°C	200	8	H_2O	H_2(5MPa)	96.1	68.1	[24]
13	NiCo/CNT	H_2/500°C	220	2	2-propanol	2-propanol	100	94	[25]
14	Co/GOr-N	H_2/300°C	300	3	dodecane	H_2(5MPa)	100	~78	[26]
15	Co/TiO_2	H_2/600°C	200	1.5	n-decane	H_2(1MPa)	100	98	[27]
16[b]	CoNx@NC-650	NH_3/650	200	1.5	dodecane	H_2(2MPa)	100	86.6	[28]

[a] GUA—guaiacol; CYHAOL—cyclohexanol. [b] Eugenol is used as feed and propylcycohexanol is the main product.

Table S2. Co-based catalysts used in hydrodeoxygenation of guaiacol [a].

Entry	Catalyst	Reduction condition	Reaction conditions				X_{GUA} mol%	$Y_{Main\ product}$ mol%	Ref.
			Temp. °C	Time h	Solvent	H_2 (MPa)			
1	Co/GOr-N	H_2/300°C	300	3	dodecane	5	100	CYHAOL (~78)	[26]
2	Co/TiO_2	H_2/600°C	200	1.5	n-decane	1	100	CYHAOL (98)	[27]
3[b]	CoNx@NC-650	NH_3/650	200	1.5	dodecane	2	100	pCYHAOL (86.6)	[28]
4	Co/ZrP	H_2/500°C	300	2.5	dodecane	4	100	CYHA (76)	[8]
5	Co/HMETS-10	H_2/400°C	280	2	dodecane	2	99.5	CYHA (96.9)	[9]
6	Co/G	H_2/300°C	300	4	dodecane	5	77	Ph (52.5)	[29]
7	CoMoS/γ-Al_2O_3	H_2/H_2S/400°C	250	-	xylene/ ethylbenzene	5.5	100	Ph (55)	[30]
8	Co-MoO_2/C	Cellulose/N_2/ 650°C	340	4	n-hexane	0.8	97	Benzene/Toluene (61.1)	[31]
9	CoMoS/γ-Al_2O_3	H_2/H_2S/400°C	350	2	n-hexadecane	8	100	Benzene (70)	[32]

[a] GUA—guaiacol; CYHAOL—cyclohexanol; CYHA–cyclohexane; Ph—phenol; pCYHAOL—propylcycohexanol. [b] Eugenol is used as feed.

3. Figures

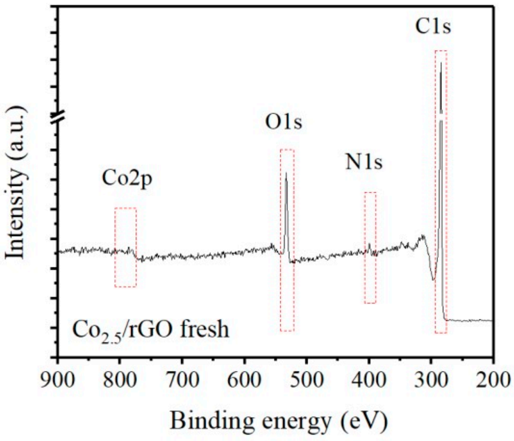

Figure S1. The full-scan XPS survey spectrum of the $Co_{2.5}$/rGO catalyst.

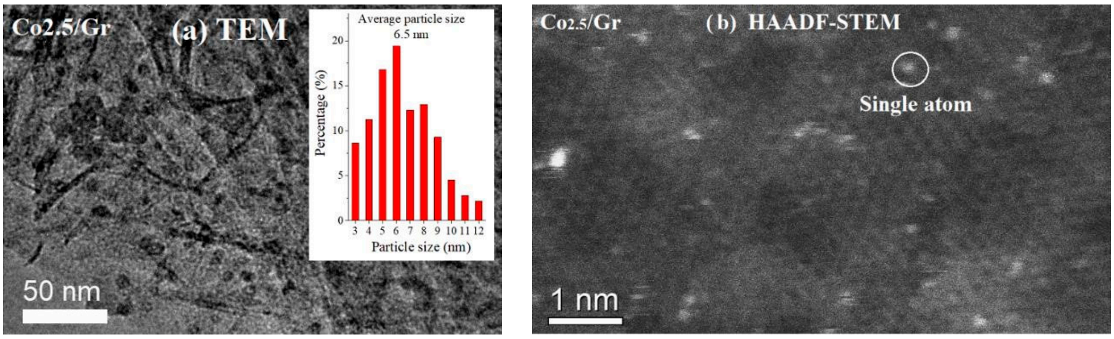

(a) **(b)**

Figure S2. Characterization results of the $Co_{2.5}$/Gr catalyst: (a) TEM image and particle size distribution; (b) HAADF-STEM image.

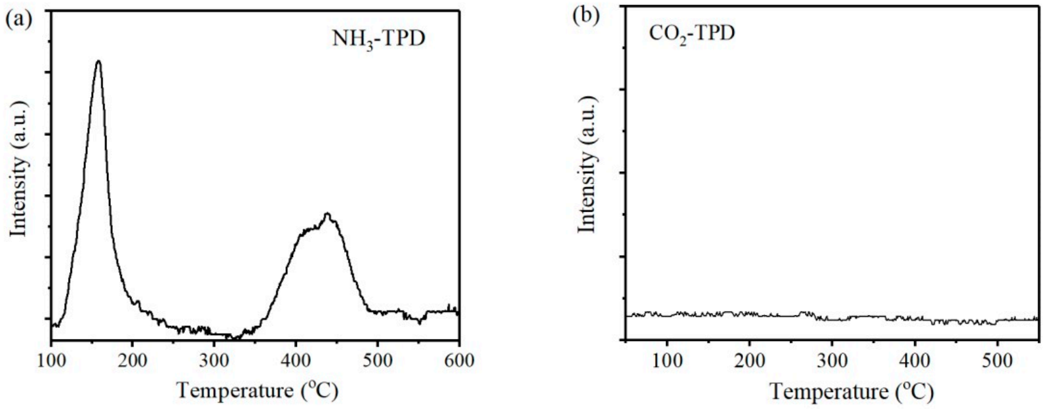

(a) **(b)**

Figure S3. The NH_3-TPD result of the CoO_x powder.
